# Spatiotemporal evolution and driving factors of eco-environmental quality in the Beijing-Tianjin-Hebei urban agglomeration in China

**DOI:** 10.1038/s41598-025-11751-y

**Published:** 2025-07-15

**Authors:** Lemeng Liu, Jinghua Long, Wei Zhang, Jintian Yang

**Affiliations:** 1 School of Public Administration, Hebei University of Economics and Business, Shijiazhuang, 050061 China; 2 Design School, Xi’ an Jiaotong-Liverpool University, Suzhou City, 215123 China; 3 Hebei Collaborative Innovation Center for Urban-Rural Integrated Development, Shijiazhuang, 050061 China

**Keywords:** RSEI, Eco-environmental quality, PLS-SEM, Driving factors, Environmental impact, Sustainability

## Abstract

This study utilized Google Earth Engine (GEE) to compute the Remote Sensing Ecological Index (RSEI) and assess the spatiotemporal evolution of eco-environmental quality in the Beijing-Tianjin-Hebei (BTH) urban agglomeration from 2000 to 2020. Additionally, Partial Least Squares Structural Equation Modeling (PLS-SEM) was used to examine how climatic, topographical, urbanization, soil, and biological factors influenced eco-environmental quality dynamics. The results showed that from 2000 to 2020, RSEI exhibited a fluctuating decline, with the proportion of areas having the highest RSEI dropping from 7.99% in 2000 to 1.20% in 2020, while regions with poor and worst RSEI levels followed a fluctuating upward trend, rising from 4.80 to 15.89%. NDVI emerged as the dominant driver of RSEI in the BTH urban agglomeration, with its contribution to the principal components peaking in 2000 and exhibiting a fluctuating downward trend thereafter until 2020. In 2015, LST turned positive in PC_1_ and similarity dropped, indicating a shift in ecological mechanisms. During this period, the ranking of key influencing factors was biological > climatic > soil > topographical > urbanization. Furthermore, the spatial distribution of RSEI exhibited distinct clustering patterns, with H–H regions mainly located in the Bashang Plateau in northern BTH, while L-L regions were concentrated in the southeastern plains, gradually expanding from scattered points to more continuous areas.

## Introduction

China’s urbanization is a significant phenomenon in global population migration and urban development^1,2^. In the past two decades, China has experienced rapid and profound urbanization, leading to increasingly severe environmental degradation^3^. The Beijing-Tianjin-Hebei (BTH) urban agglomeration, as a paradigmatic illustration of China’s process of urbanization and the third largest urban agglomeration after the Yangtze River Delta and the Pearl River Delta, has become one of the most economically dynamic, densely populated, and highly urbanized regions in northern China^4–6^. However, the overall development situation of the Beijing-Tianjin-Hebei region still faces some problems compared with that of the Yangtze River Delta region and the Pearl River Delta region^8^. The very dynamism has accelerated severe air pollution, chronic water scarcity, and widespread ecosystem disruption^9–11^. During the 2001–2008 preparatory period for the 29th Summer Olympic Games and the lead-up to the 2014 APEC Summit, the BTH region not only pursued economic integration but also piloted a series of environmental protection policies. In particular, authorities promulgated “the Joint Regional Air Pollution Control Policy” and “the Action Plan for Air Pollution Prevention and Control in Beijing-Tianjin-Hebei and Surrounding Areas”. Empirical evaluations of these measures have confirmed that coordinated, region-wide development exerts a measurable, positive impact on ecological quality^12^. Hence, the “Beijing-Tianjin-Hebei Coordinated Development Plan” was released in 2015^13^. It clearly outlined the strategy of coordinated development for the BTH urban agglomeration, aiming for deep integration and balanced development in areas such as the economy, society, and environment^14,15^. This coordinated development is expected to have a profound influence on the regional eco-environment^13^. Therefore, evaluating the eco-environmental quality in the BTH urban agglomeration and exploring its driving forces are crucial for assessing the implementation of policies, balancing economic growth with ecological conservation and promoting high-quality urbanization.

The methods used to evaluate eco-environmental quality include the Pressure-State-Response (PSR) model, Ecosystem Health Assessment Framework (EHAF), Comprehensive Ecological Rsik Index (CERI), and Ecological Carrying Capacity Analysis (ECCA)^16–19^. Advancements in remote sensing technology have provided essential support for the accurate and efficient assessment of ecological environments. Previous studies have largely relied on individual remote sensing data, such as the Normalized Difference Vegetation Index (NDVI), Enhanced Vegetation Index (EVI), and Land Surface Temperature (LST)^20–22^. However, due to the complexity of ecosystems and the diversity of influencing factors, a single indicator cannot fully capture the intricate ecological conditions^23^. As a result, comprehensive evaluation methods are necessary. Common methods used in earlier studies include the Ecological Environment Status Index (EI) and the Remote Sensing Ecological Index (RSEI)^24,25^. The RSEI, which utilizes remote sensing data, evaluates eco-environmental quality through Principal Component Analysis (PCA) of indicators such as greenness, humidity, temperature, and dryness^26^. By integrating multiple indicators, the RSEI is better equipped to reflect the subtle responses of ecosystems to environmental changes, offering more comprehensive feedback on eco-environmental quality. The data used for this method are easily accessible, timely, and effective for assessment across various spatial scales. Although the RSEI is now widely applied and can underpin subsequent analyses, such as geographic detectors and PLS-SEM models, some researchers warn that its uncritical use without adaptive validation may lead to distorted results^27^. For example, in certain study areas it is necessary to compare weighting schemes, such as PCA and the entropy weighting method (EWM), to identify the most appropriate assignment for RSEI^28^. Moreover, in specialized contexts (e.g., urban centers, mining zones, or high-altitude regions), the presence of impervious concrete, bare soil, or snow can prevent the original indicators from accurately reflecting ecological quality; in such cases, local calibration or the incorporation of auxiliary indices tailored to site characteristics is required to develop some improved RSEI methods for specific purposes^29–32^. Finally, because RSEI is highly sensitive to surface phenology, and few studies have determined the optimal observation window for specific regions, its application still demands further in-depth investigation^33,34^.

Various factors could influence the eco-environmental quality, including natural, geographical, socioeconomic, and biological factors. Natural & geographical factors include climate (precipitation, temperature, etc.), soil (sand, clay, soil organic carbon, etc.), and terrain (elevation, slope, etc.). Socio-economic factors include population (population density), urbanization (land cover classification, normalized difference built-up index, etc.), and Gross Domestic Product (GDP). Biological factors typically involve indicators such as NDVI, Gross Primary Productivity (GPP), or Above-ground Biomass (AGB). Previous research used methods such as correlation, cluster, and multiple regression analyses to analyze the driving factors of eco-environmental quality. However, these methods focus only on the relationship between individual factors and eco-environmental quality, making it difficult to accurately express the magnitude of the relationships between the variables. Partial least squares structural equation Modeling (PLS-SEM) can simultaneously process multiple variables, accurately observe the strength of the relationships between them, and analyze the indirect paths and causal relationships among various factors. Moreover, PLS-SEM requires fewer samples and does not require normally distributed data. By combining PLS-SEM with geographic detector technology, it is possible to gain a deeper understanding of the underlying patterns of regional eco-environmental quality changes and explore their driving factors.

To explore the response of eco-environmental quality to natural and human factors during urbanization, we used the Google Earth Engine (GEE) platform to calculate the RSEI. With a cycle of five years, it analyzes the spatiotemporal variations of eco-environmental quality in the BTH urban agglomeration from 2000 to 2020, aiming to test the implementation effect of each “five-year Plan” of China^35^, and introduces PLS-SEM to analyze the impact mechanisms of climate, terrain, urbanization, soil, and biological factors on the shifts in eco-environmental quality within the region. This study achieves some contributions, which are as follows: (1) the use of GEE to compute the RSEI based on PCA and (2) the inclusion of GPP and AGB as biological factors, in addition to natural, geographical, and socioeconomic factors, which helps gain a more comprehensive understanding of the influence of biological factors on regional eco-environmental quality.

## Materials and methods

### Study area

**Fig. 1:**
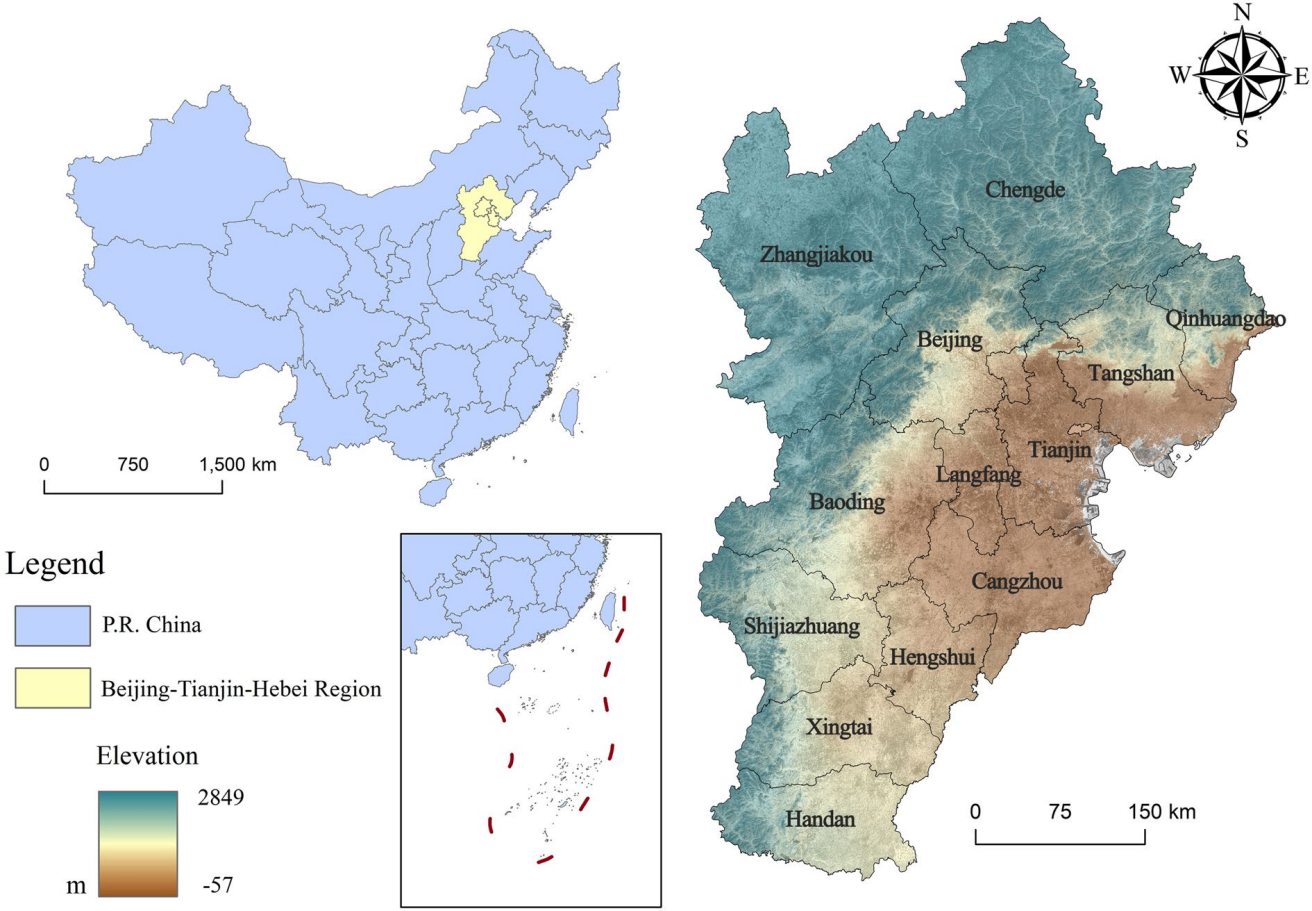
Location map of BTH urban agglomeration. The map is created using ArcGIS 10.8 (https://desktop.arcgis.com/en/arcmap/latest/get-started/main/get-started-with-arcmap.htm). The sdministrative boundary vector of the study area are sourced from the Environmental Science and Data Center, CAS (https://www.resdc.cn/). The color satellite image and the Digital Elevation Model (DEM) are from the Sentinal-2 dataset (https://code.earthengine.google.com/7719dbc4cd4e85fe2c7f9658d45ea94f) and the MODIS terrain database (https://code.earthengine.google.com/cbee1df6686a326ee1459189782879b9) of Google Earth Engine, respectively.

The Beijing-Tianjin-Hebei (BTH) urban agglomeration is located in northern China and encompasses the municipalities of Beijing, Tianjin, and Hebei Province (Fig. 1), covers an area of approximately 216,700 km^2^ and has a population of approximately 110 million. The region has a warm temperate monsoon climate, with average annual temperatures between 14 and 15 °C and annual precipitation between 500 and 800 mm. The terrain in the BTH urban agglomeration is diverse, featuring the Yan and Taihang Mountains, and hosts a variety of vegetation types, including forests, grasslands, and agricultural lands, which together form a range of ecosystems.

### Data source

**Fig. 2:**
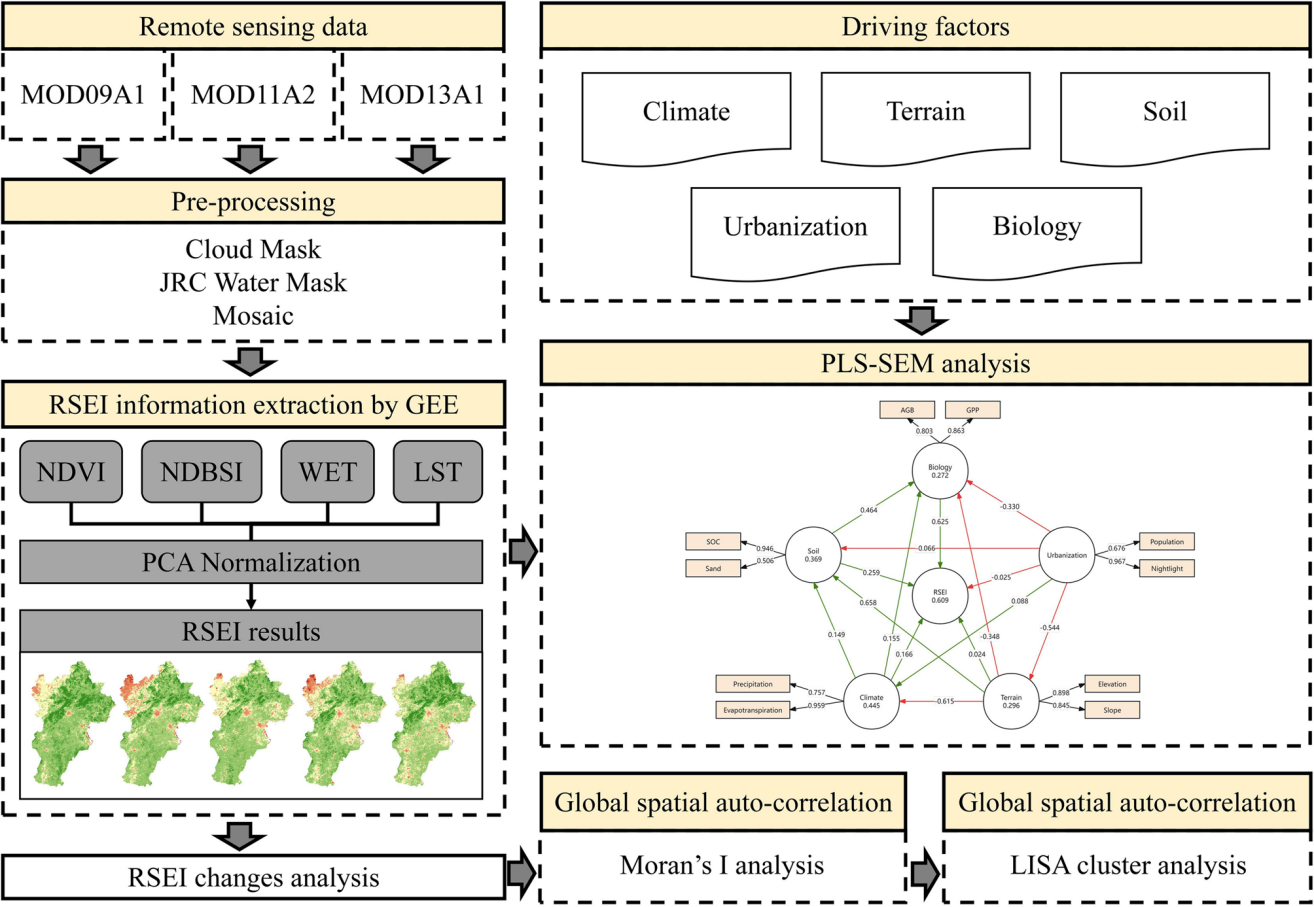
Technical flow chart of the study.

In this study, RSEI of the BTH urban agglomeration from 2000 to 2020 were sourced from the MOD09A1, MOD11A2, and MOD13A1 datasets on GEE platform, which included data on LST, NDVI, Normalized Difference Built-up Index (NDBSI), and WET. To ensure the accuracy of the data, the study selected June 1st to August 31st as the observation period for the RSEI in the BTH urban agglomeration based on prior studies^33^. Precipitation and evapotranspiration serve as key climate indicators, while terrain characteristics, such as Digital Elevation Model (DEM) and slope, provide insights into the landform. Soil indicators includes sand and soil organic carbon (SOC), economic indicators include population and nightlight data, while AGB and GPP reflect biological conditions (Table 1). The detailed flow chart is shown in Fig. 2.

**Table 1:** Datasets catalog introduction.

Data	Resolution	Source
Administrative boundary vector	–	The Environmental Science and Data Center, Chinese Academy of Sciences (https://www.resdc.cn/)
RSEI	1 km	MOD09A1, MOD11A2, and MOD13A1 databases, Google Earth Engine (https://code.earthengine.google.com/86e4885f482e7ee7ae26364f1071da1b)
Precipitation		The National Earth System Science Data Center of China (https://www.geodata.cn/data/datadetails.html?dataguid=113786088533256docId=6)
Evapotranspiration		MOD16A2 database, Google Earth Engine (https://code.earthengine.google.com/454c0843c5f68418e887d6e8ef72b2cb)
DEM		MODIS terrain database of Google Earth Engine (https://code.earthengine.google.com/cbee1df6686a326ee1459189782879b9)
Slope		Soil Database of the United Nations Food and Agriculture Organization (https://www.fao.org/soils-portal/data-hub/soil-maps-and-databases/harmonized-world-soil-database-v12/en/)
Soil		
Population		Landscan database (https://landscan.ornl.gov/)
Nighlight data		The Harvard University Nightlight Database^36^
AGB		PIE Remote Sensing Computing Service Platform (PIE-RSCSP) (https://engine.piesat.cn/engine-share/shareCode.html?id=77e8a94657ff477582835c7dd4b26b4c)
GPP		The National Tibetan Plateau Scientific Data Center^37^

## Methods

### RSEI assessment framework

The RSEI evaluates the eco-environmental quality through four ecological indicators: greenness, humidity, temperature, and dryness^38^. The calculation formula is:

$$RSEI=f(NDVI, WET, LST, NDBSI)$$

where *NDVI* represents greenness, *WET* indicates humidity, *LST* stands for temperature, and *NDBSI* corresponds to dryness.

In this study, a water body mask was used to avoid interference of the water surface with the principal components, and the indicators were normalized before performing the PCA^39^. The normalization formula is:

$$N=\frac{V-{V}_{min}}{{V}_{max}-{V}_{min}}$$

where *N* is the normalized value of a metric, while *V* is the value of the metric within a given pixel.

The NDVI formula is^40^:

$$NDVI=\frac{{\rho }_{NIR}-{\rho }_{Red}}{{\rho }_{NIR}+{\rho }_{Red}}$$

where *ρ*_NIR_ denotes the reflectance in the near-infrared band, while *ρ*_Red_ signifies the reflectance in the red band.

The WET formula is^41^:

$$WET = \delta 1\rho Blue + \delta 2\rho Green + \delta 3\rho Red + \delta 4\rho NIR + \delta 5\rho SWIR1 + \delta 6\rho SWIR2$$

where *δ*1 − *δ*6 denote the reflectance values for each respective band of the remote sensing image, and *ρ*_Blue_, *ρ*_Green_, *ρ*_Red_, *ρ*_NIR_, *ρ*_SWIR1_, and *ρ*_SWIR2_ denote the values of the corresponding bands.

The LST formula is^42^:

$$LST=\frac{T}{1 + (\lambda T/\rho ) \times ln\varepsilon }-273.15$$

where *T* is degrees Celsius (°C), *λ* is the wavelength of the emitted radiation (10.895 µm for the band 10), $\varepsilon$ is the surface emissivity, h is Planck’s constant (6.626 × 10–34 J·s), *c* is the speed of light (2.998 × 108 m/s), and *σ* is Boltzmann’s constant (1.38 × 10–23 J/K).

The NDBSI formula is^43^:

$$NDBSI=\frac{(SI + IBI)}{2}$$

$$SI=\frac{({\rho }_{SWIR1}+{\rho }_{Red})-({\rho }_{NIR}+{\rho }_{Blue} )}{({\rho }_{SWIR1}+{\rho }_{Red})+({\rho }_{NIR}+{\rho }_{Blue} )}$$

$$IBI=\frac{\frac{2\times {\rho }_{SWIR1}}{{\rho }_{SWIR1}{+\rho }_{NIR}}-(\frac{{\rho }_{NIR}}{{\rho }_{NIR}+{\rho }_{Red}}+\frac{{\rho }_{Green}}{{\rho }_{Green}+{\rho }_{SWIR1}})}{\frac{2\times {\rho }_{SWIR1}}{{\rho }_{SWIR1}{+\rho }_{NIR}}+(\frac{{\rho }_{NIR}}{{\rho }_{NIR}+{\rho }_{Red}}+\frac{{\rho }_{Green}}{{\rho }_{Green}+{\rho }_{SWIR1}})}$$

where *SI* denotes the soil index, *IBI* denotes the building index, and *ρ*_Blue_, *ρ*_Green_, *ρ*_Red_, *ρ*_NIR_, and *ρ*_SWIR1_ represent the reflectance values of the corresponding bands.

Subsequently, PCA was used to calculate PC_1_^38^:

$$RSEI={PC}_{1}\left[f\left(NDVI, WET, LST, NDSI\right)\right]$$

where *PC*_*1*_ represents the first principal component, and *f* denotes the normalization of each indicator.

We categorize the RSEI into five levels in this study: worst (0–0.2), poor (0.2–0.4), moderate (0.4–0.6), good (0.6–0.8), and excellent (0.8–1.0).

### Cosine similarity

Cosine Similarity (CS) measures the cosine of the angle between two vectors and is used to assess the directional similarity between principal component loading vectors. This method is commonly applied to compare principal components across years, with results ranging from − 1 to 1. A CS value close to 1 indicates that the two vectors are nearly in the same direction, reflecting a high degree of structural stability of the corresponding principal component^44^. If the CS between two components is 0.85–0.94, the two components can be considered fairly similar to each other. If the CS is equal to or greater than 0.95, the two components can be considered remarkably similar^45^.

$$Cos\alpha =\frac{a\cdot b}{\parallel a\parallel \cdot \parallel b\parallel }$$

Here, $a$ and $b$ represent two principal component loading vectors, *a* $\cdot$ *b* denotes the vector dot product, $\parallel a\parallel$ and $\parallel b\parallel$ are the Euclidean norms of the vectors.

### PLS-SEM structural equation modelling

PLS-SEM is a component-based estimation technique primarily used to analyze causal relationships within path models containing latent variables. It alternately and iteratively estimates the measurement model (i.e., the relationships between latent constructs and their observed indicators) and the structural model (i.e., the paths among the latent constructs), with the objective of maximizing the explained variance of the endogenous variables^46^. PLS-SEM consists of measurement and structural models, which are used to study latent variables and explore the mechanisms of action between them^47^. PLS-SEM does not require multivariate normality of the data and remains robust even with relatively small sample sizes or high model complexity^48^. The formulae are as follows:

$$x={\bigwedge }_{x}\xi +\delta$$

$$y={\bigwedge }_{y}\eta +\varepsilon$$

where *x* represents a vector of exogenously observed variables, and *y* denotes a vector of endogenously observed variables. Λ*x* and Λ*y* are matrices of factor loadings from *ξ* to *x* and from *η* to y, respectively. Meanwhile, *δ* and *ε* represent vectors of measurement error. The relationship between these two latent variables is expressed through the following regression equation:

$$\eta =B\eta +\Gamma \xi +\zeta$$

where *η* denotes a vector of endogenous latent variables, and *ξ* represents a vector of exogenous latent variables. *B* is the regression path coefficient describing the relationships among different *η* variables, while *Γ* represents the regression path coefficient capturing the influence of *ξ* on *η*. Lastly, *ζ* accounts for the residuals in the model measurement.

In this study, the following variables were selected as significant factors: precipitation and temperature as climatic factors; sand and soil organic carbon content as soil factors; elevation and slope as topographic factors; night light index and population density as social factors; and total primary productivity and surface biomass as biological factors.

### Spatial auto-correlation

Spatial autocorrelation reflects the spatial dependence and heterogeneity of specific indicators due to geographic location or neighborhood and is used to measure the spatial distribution structure of indicators in a regional system^49^. In this study, based on the global spatial autocorrelation test for spatial clustering, we applied local spatial autocorrelation (LISA) analysis (Local Moran’s I Analysis) to identify the spatial clustering pattern of eco-environmental quality in the BTH urban agglomeration^50^, and used the Moran’s I index to reflect the spatial clustering pattern of RSEI^51^.

Global spatial auto-correlation:

$${I}_{Global}=\frac{N{\sum }_{i}\sum_{j}{W}_{ij}({x}_{i}-\mu )({x}_{j}-\mu )}{({\sum }_{i}\sum_{j}{W}_{ij}{){\sum }_{i}({x}_{i}-\mu )}^{2}}$$

where *n* denotes the number of administrative regions, and W_ij_ denotes the spatial weight matrix. *x*_i_ and *x*_j_ correspond to the RSEI values within each grid, while $\mu$ represents the mean RSEI value, and *N* is the total number of grids. A positive value of the indicator (${I}_{Global}$> 0) suggests a positive spatial correlation with habitat quality in the BTH urban agglomeration, whereas a negative value (${I}_{Global}$< 0) indicates a negative spatial correlation with eco-environmental quality.

Local spatial auto-correlation:

$${I}_{Local}=\frac{{x}_{i}-\mu }{{\sum_{i}({x}_{i}-\mu )}^{2}}\sum_{i\ne j}{W}_{ij}({x}_{i}-\mu )$$

Based on the calculation results, the area can be categorized into four regions: high-high (H–H), high-low (H–L), low–high (L–H), and low-low (L-L). When ${I}_{Local}$ >0, H–H or L-L indicates minimal variation in eco-environmental quality, meaning both the area and its neighboring regions exhibit consistently high or low quality. Conversely, when ${I}_{Local}$ <0, L–H or H–L suggests a significant disparity in urbanization levels, implying that the eco-environmental quality of the area is either higher or lower than that of its surroundings. A value of ${I}_{Local}$ =0 indicates an insignificant difference in eco-environmental quality.

## Results

### Characteristics of spatial and temporal evolution of RSEI in BTH

The results obtained after standardizing the loadings and the CS of PC_1_ loadings are summarized in Table 2 and Table S2.

**Table 2:** Loading matrix and PC_1_ contribution ratio of four RSEI indicators in BTH (2000–2020).

Year	Indicators	NDVI	LST	WET	NDBSI	Percentage
2000	PC_1_	0.9689	− 0.2042	0.1346	0.0387	65.44%
	PC_2_	− 0.1869	− 0.2528	0.9482	0.0462	21.40%
	PC_3_	0.1586	0.9456	0.2815	0.0392	13.05%
	PC_4_	− 0.0352	− 0.0175	− 0.0602	0.9974	0.10%
2005	PC_1_	0.8708	− 0.2961	− 0.3908	− 0.0377	74.08%
	PC_2_	− 0.2905	0.3336	− 0.8958	− 0.0437	15.92%
	PC_3_	0.3948	0.8938	0.2072	− 0.0483	9.94%
	PC_4_	− 0.0393	− 0.0468	0.0440	− 0.9972	0.05%
2010	PC_1_	0.9228	− 0.0772	0.3760	− 0.0337	68.98%
	PC_2_	− 0.3255	0.3672	0.8703	− 0.0432	17.16%
	PC_3_	0.2041	0.9242	− 0.3167	− 0.0624	13.76%
	PC_4_	− 0.0299	− 0.0712	− 0.0306	− 0.9965	0.10%
2015	PC_1_	0.8855	0.0695	− 0.4585	− 0.0277	65.00%
	PC_2_	− 0.3209	− 0.6233	− 0.7127	− 0.0236	22.61%
	PC_3_	0.3342	− 0.7770	0.5309	− 0.0531	12.33%
	PC_4_	− 0.0348	0.0541	0.0014	− 0.9979	0.06%
2020	PC_1_	0.8559	− 0.3019	0.4189	− 0.0301	63.33%
	PC_2_	− 0.4512	− 0.8326	0.3191	− 0.0384	23.52%
	PC_3_	0.2521	− 0.4603	− 0.8500	− 0.0463	13.08%
	PC_4_	− 0.0201	0.0626	0.0145	− 0.9977	0.07%

From 2000 to 2020, the contribution rates of PC_1_ were 65.44, 74.08, 68.98, 65.00, and 63.33%, respectively, demonstrating that PC_1_ accounted for the majority of the characteristic information from all indicators. Meanwhile, Jin et al. (2025) demonstrated the rationality of the application of the RSEI model based on PC_1_ in the Beijing-Tianjin-Hebei region in their research^52^. Compared with PC_1_, the contribution rates of other principal components are all lower than 30%, indicating that PC_1_ integrates most of the characteristics of the four ecological indicators, making it a feasible remote sensing ecological indicator^53,54^. Moreover, the CS of PC_1_ loadings consistently exceeded 0.85 for most of the period from 2000 to 2020, in contrast, the structure of the loading vector matrix underwent significant declined to 0.7810 in 2015. Meanwhile, the eigenvalues (EV) of PC_1_ are stable in each year, which indicates that the structure of PC_1_ remained highly stable^24^. Moreover, NDVI exhibited a positive correlation with PC_1_, whereas LST correlated negatively, a pattern that aligns with fundamental ecological principles^55^. In conclusion, the dominant contribution rate, high stability, consistent structure of PC_1_, and ecologically meaningful indicator loadings collectively support the reliability and scientific validity of the PCA-based weighting method employed in this study.

From 2000 to 2020, NDVI consistently had the highest positive loading on PC_1_, with values of 0.9689, 0.8708, 0.9228, 0.8855, and 0.8559 respectively, indicating its persistent positive influence on eco-environmental quality. Conversely, LST showed a predominantly negative contribution to PC_1_, underscoring its generally adverse effect on regional ecological quality.

Notably, the cosine similarity of PC_1_ loadings between 2015 and other years showed a marked decline (minimum value of 0.781), significantly below the standard threshold of 0.85 for structural comparability. Meanwhile, LST shifted from negative to positive. This indicates that in 2015, there were substantial ecological or environmental shifts potentially linked to major socio-economic transitions, industrial restructuring, or specific climatic anomalies. Despite this variability, the eigenvalue (EV) of PC_1_ across all years remained relatively stable (ranging from 0.0135 to 0.0175), suggesting that the proportion of variance explained by PC_1_ was consistent over time, indicating robust dimensional stability and reducing potential scale bias introduced by differences in PCA contribution rates.

In summary, while NDVI, a key determinant of regional ecological quality, consistently showed an antagonistic relationship with LST throughout the study period, substantial variations in principal component loadings, particularly in 2015, emphasize the need to consider the ecological and socio-economic conditions unique to each year when interpreting the results. These findings also call for careful interpretation when comparing PCA outcomes across different years and highlight the importance of identifying the underlying factors driving such changes.

**Fig. 3:**
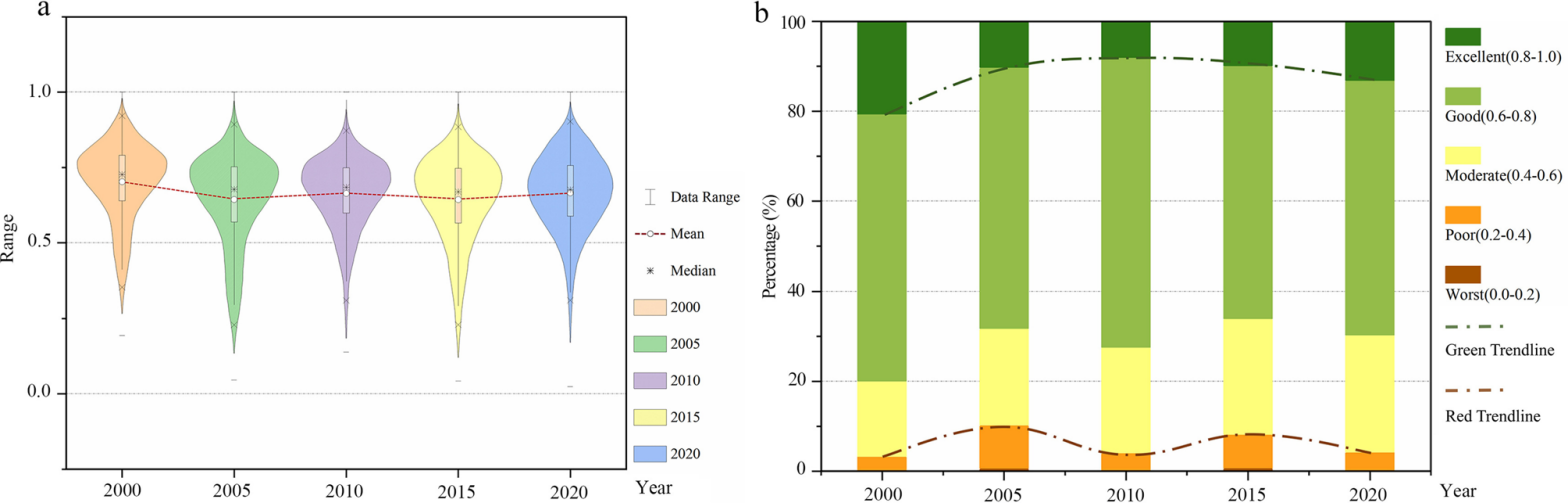
Box plot of RSEI for BTH urban agglomeration and percentage of regions at each level (2000–2020). The figure is created using Origin 2024 (https://www.originlab.com/2024). (**a**) The figure is generated by applying the sampling tool of ArcGIS 10.8 to the RSEI data. (**b**) The figure is generated using sampled RSEI data that have been reclassified.

As shown in Fig. 3a, between 2000 and 2020, the RSEI in the BTH urban agglomeration exhibited a fluctuating downward trend. Specifically, the RSEI values for the years 2000, 2005, 2010, 2015, and 2020 were 0.695, 0.637, 0.661, 0.638, and 0.660, respectively.

The RSEI of this study is divided into five parts, and this classification method was first proposed by Xu et al. (2018)^38^. From Fig. 3b, it can be observed that the proportion of areas with “Excellent” RSEI in the BTH urban agglomeration for the years 2000, 2005, 2010, 2015, and 2020 were 20.66, 10.29, 8.09, 9.92, and 13.23%, respectively. From 2000 to 2020, the ecologically high-quality areas marked by the green trend line exhibited a fluctuating downward trend, decreasing from 20.66% in 2000 to 13.23% in 2020, with the lowest value occurring in 2010 (8.09%). Conversely, the area of ecological damage, marked by a red trend line, shows a fluctuating upward trajectory, increasing from 3.18% in 2000 to 4.15% in 2020, peaking in 2005 (10.15%).

**Fig. 4:**
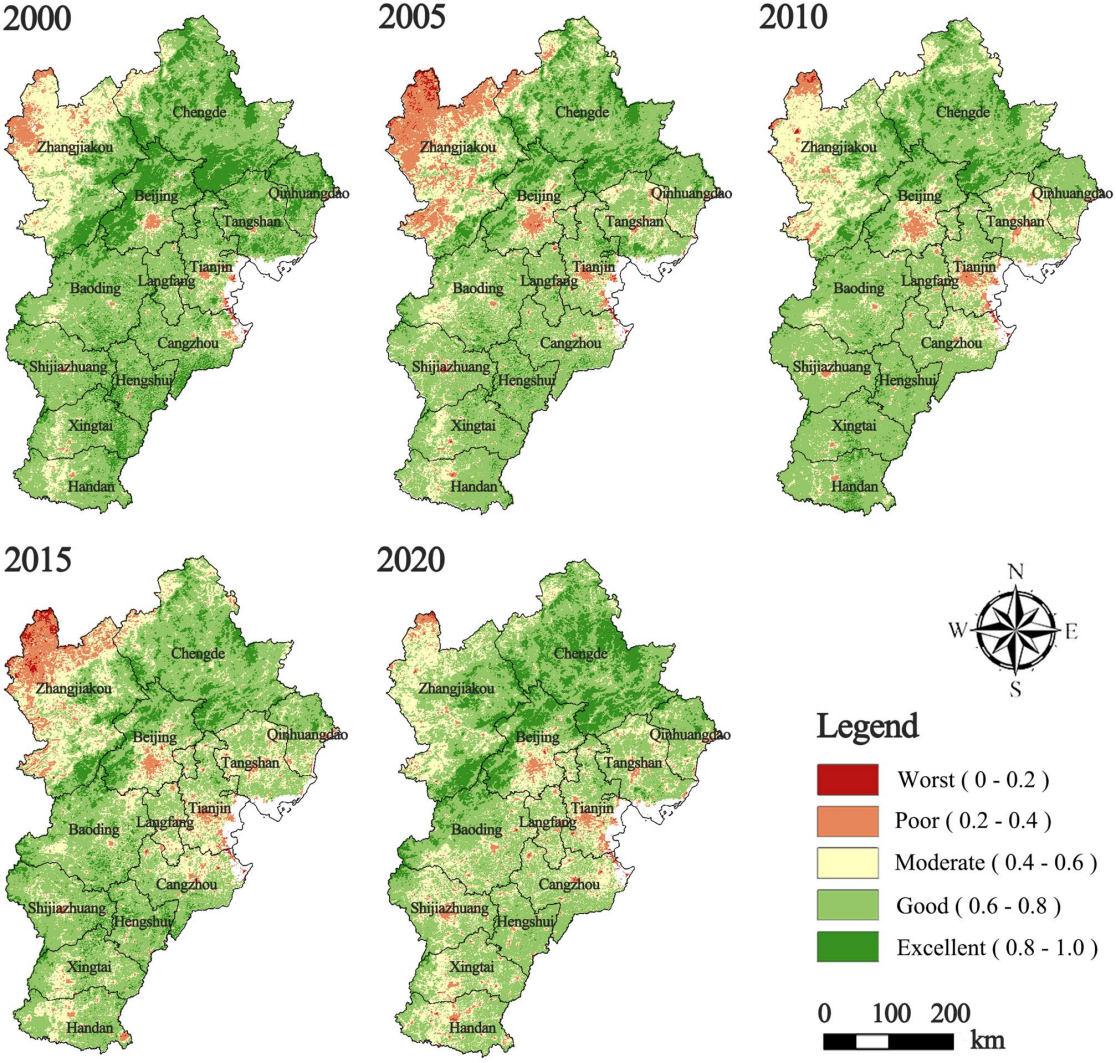
Spatial distribution of RSEI in BTH urban agglomeration (2000–2020). The spatial distribution of RSEI is calculated using Google Earth Engine (https://code.earthengine.google.com/e207df6e0205bd85e0dbe0875db564d4), and the resulting figure is edited in ArcGIS 10.8 using reclassification tool.

Between 2000 and 2020, notable changes in the RSEI of the BTH urban agglomeration were observed (Fig. 4). During this period, ecological degradation was predominantly concentrated in western Zhangjiakou and the central urban districts of Beijing, Tianjin, and Tangshan. From 2000 to 2005, overall ecological quality in the region deteriorated markedly, with degraded areas expanding significantly, especially around northwestern Zhangjiakou and Tangshan. During this period, industrialization in Beijing accelerated, and restoration projects such as the “Beijing-Tianjin Sandstorm Source Control Project” were still under construction, yielding minimal ecological benefits. Between 2005 and 2010, most areas experienced pronounced ecological recovery, particularly in Zhangjiakou, Chengde, and the northern periphery of the BTH region, resulting in a reduction in severely degraded zones, in agreement with the conclusions of Xu et al. (2018)^38^. However, from 2010 to 2015, a clear reversal occurred. Degraded areas expanded again in northwestern Zhangjiakou and its surroundings, transforming from isolated patches into extensive, contiguous degradation belts. Although regional policies such as “the Joint Regional Air Pollution Control Policy” and “the Action Plan for Air Pollution Prevention and Control in Beijing-Tianjin-Hebei and Surrounding Areas” were implemented, their ecological benefits may have been offset by concurrent adverse climatic conditions. From 2015 to 2020, ecological conditions in the BTH region improved once more, notably in Zhangjiakou, Chengde, and the outer suburbs of the BTH area. Simultaneously, Phase I of “the Beijing-Tianjin Sandstorm Source Control Project” was completed, and Phase II was initiated, yielding significant ecological restoration outcomes.

**Fig. 5:**
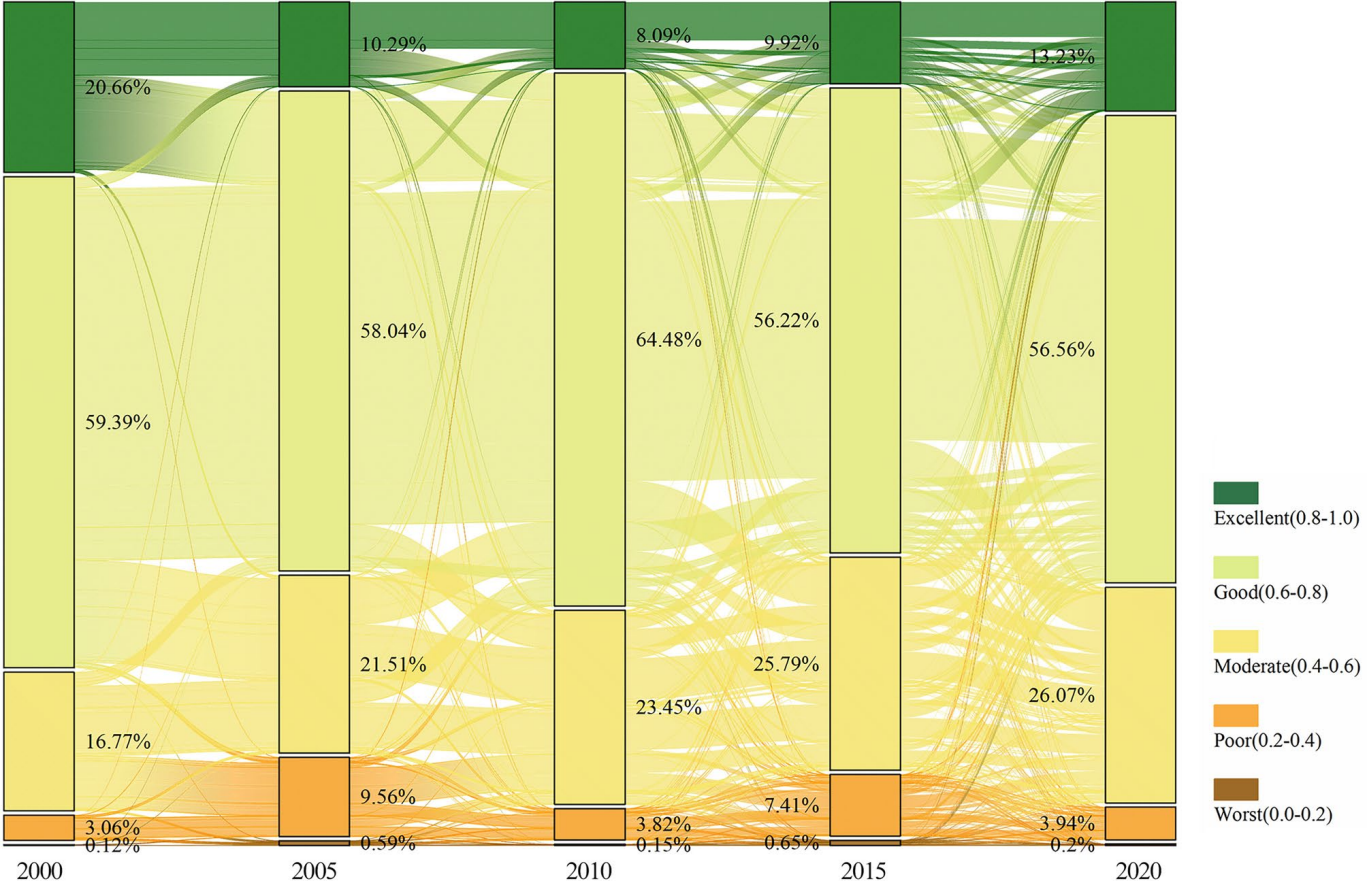
RSEI transfer in the BTH urban agglomeration (2000–2020). The figure is created using Origin 2024 (https://www.originlab.com/2024), its data are extracted from the reclassified RSEI dataset using the sampling tool in ArcGIS 10.8.

As shown in Fig. 5, the overall RSEI of the BTH urban agglomeration exhibited a downward trend from 2000 to 2005. The largest transition was from “Good” to “Moderate”, with a transfer amount of 34,436 (12.33%), covering an area of approximately 26,643.11 km^2^. At the same time, the transfer from “Excellent” to “Good” was 31,675 (11.34%), covering an area of about 24,506.93 km^2^, while the shift from “Moderate” to “Poor” was 17,838 (6.39%), occupying approximately 13,801.25 km^2^.

From 2005 to 2010, the RSEI of the region showed significant recovery. The most crucial shift was from “Moderate” to “Good”, with a transfer amount of 23,564 (8.44%), covering an area of approximately 18,231.45 km^2^. The transfer from “Poor” to “Moderate” was 17,149 (6.14%), measuring about 13,268.17 km^2^.

From 2010 to 2015, the RSEI continued to improve. The most remarkable shift was again from “Moderate” to “Good”, with a transfer amount of 27,571 (18.06%), covering an area of approximately 21,331.66 km^2^. Additionally, the shift from “Good” to “Excellent” was 30,409 (10.88%), spanning around 23,527.42 km^2^.

The RSEI of the region has declined significantly from 2015 to 2020. The most notable shift was from “Moderate” to “Good” with a transfer amount of 28,885 (10.35%), taking up about 22,348.30 km^2^. There was also a shift from “Good” to “Moderate” (21,207, 7.60%, covering 16,407.84 km^2^) and from “Poor” to “Moderate” (11,296, 6.64%, covering 8,739.71 km^2^).

**Fig. 6:**
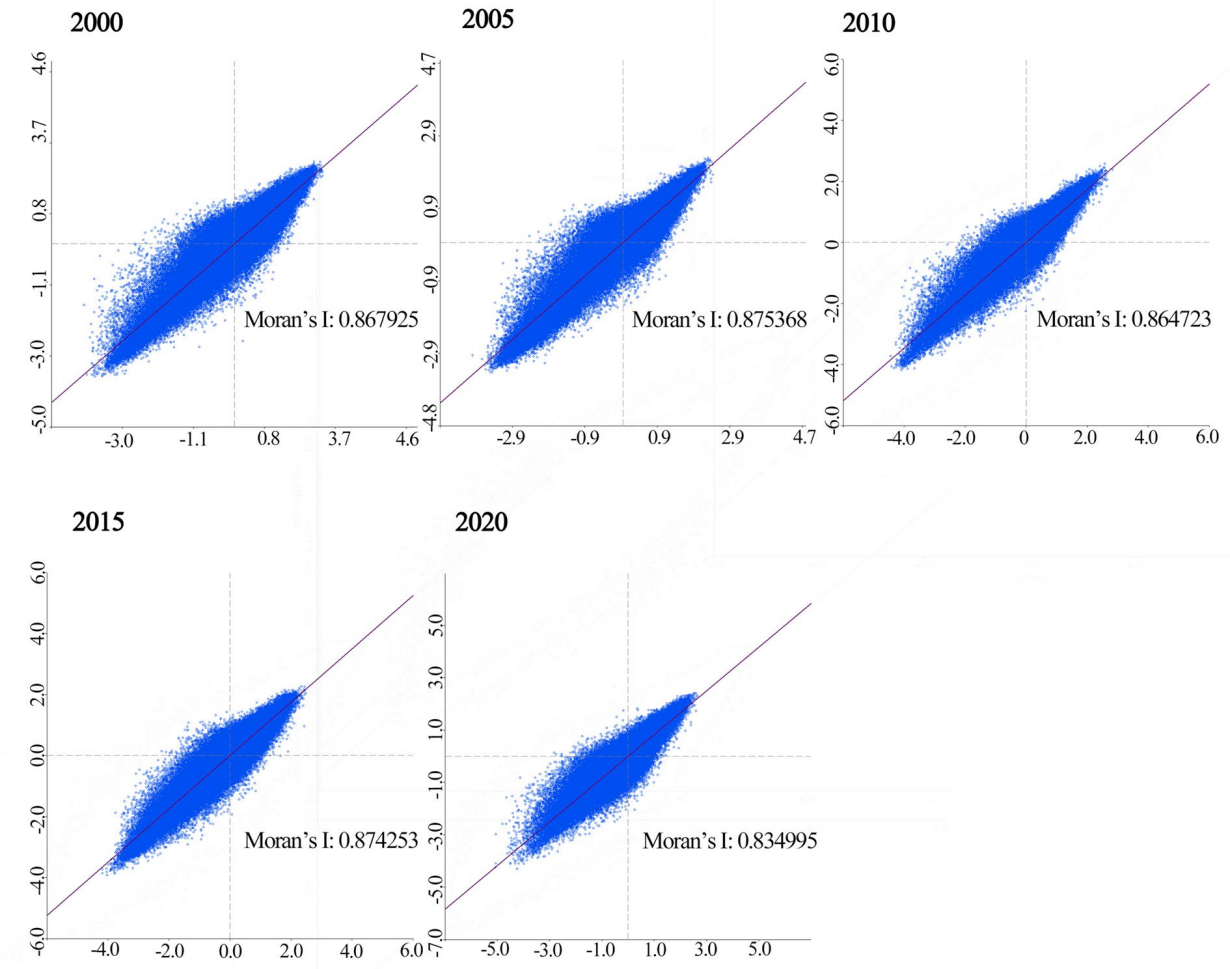
Scatter plot of RSEI Moran’s Index for the BTH urban agglomeration. The figure is generated in GeoDa 1.22.0.4 (https://geodacenter.github.io/download.html) by applying the Univariate Moran’s I tool to RSEI data sampled in ArcGIS 10.8.

**Fig. 7:**
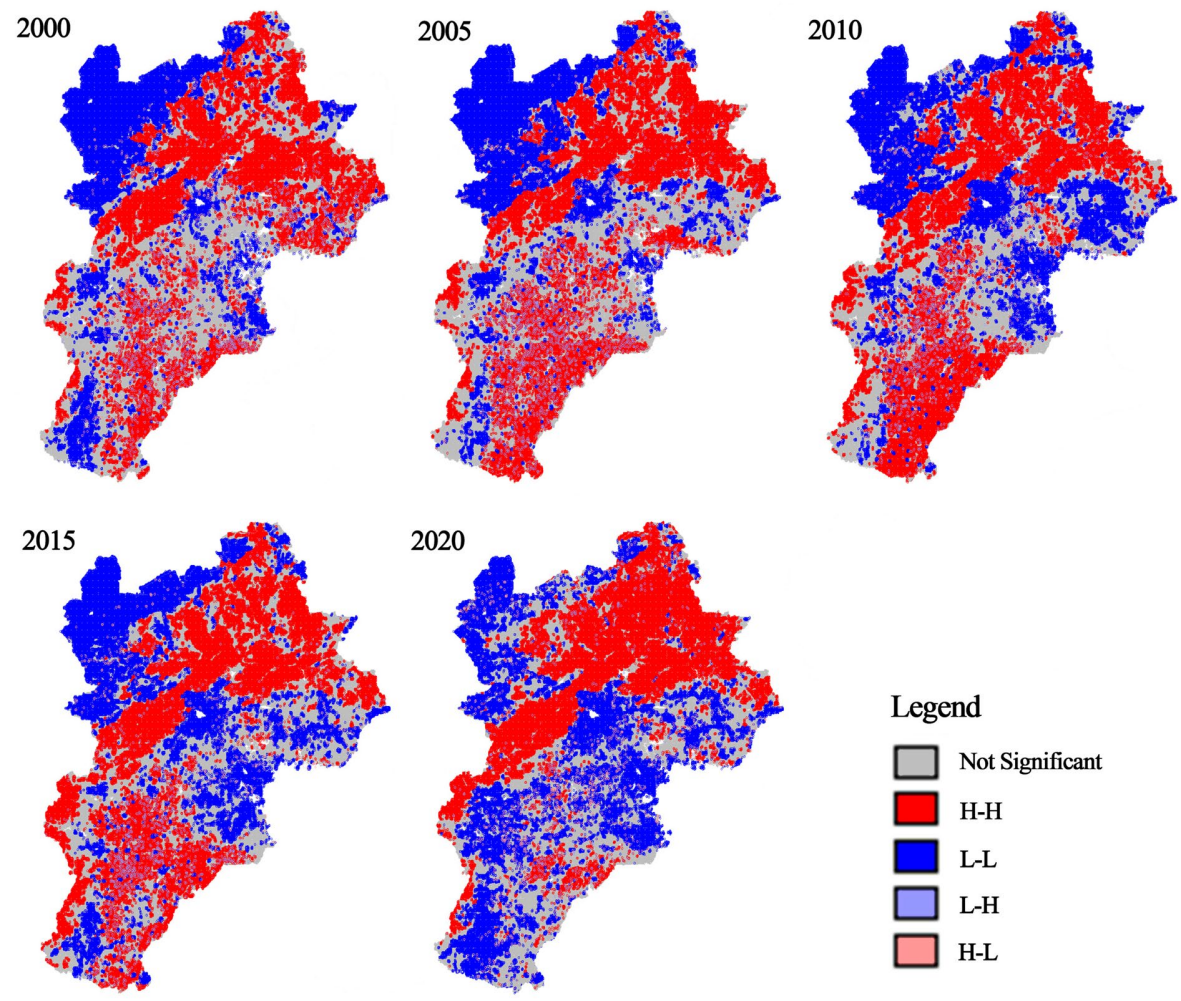
LISA clustering of RSEI in the BTH urban agglomeration. The figure is created in GeoDa 1.22.0.4 using the Univariate Local Moran’s I clustering plot option on RSEI data sampled in ArcGIS 10.8.

**Fig. 8:**
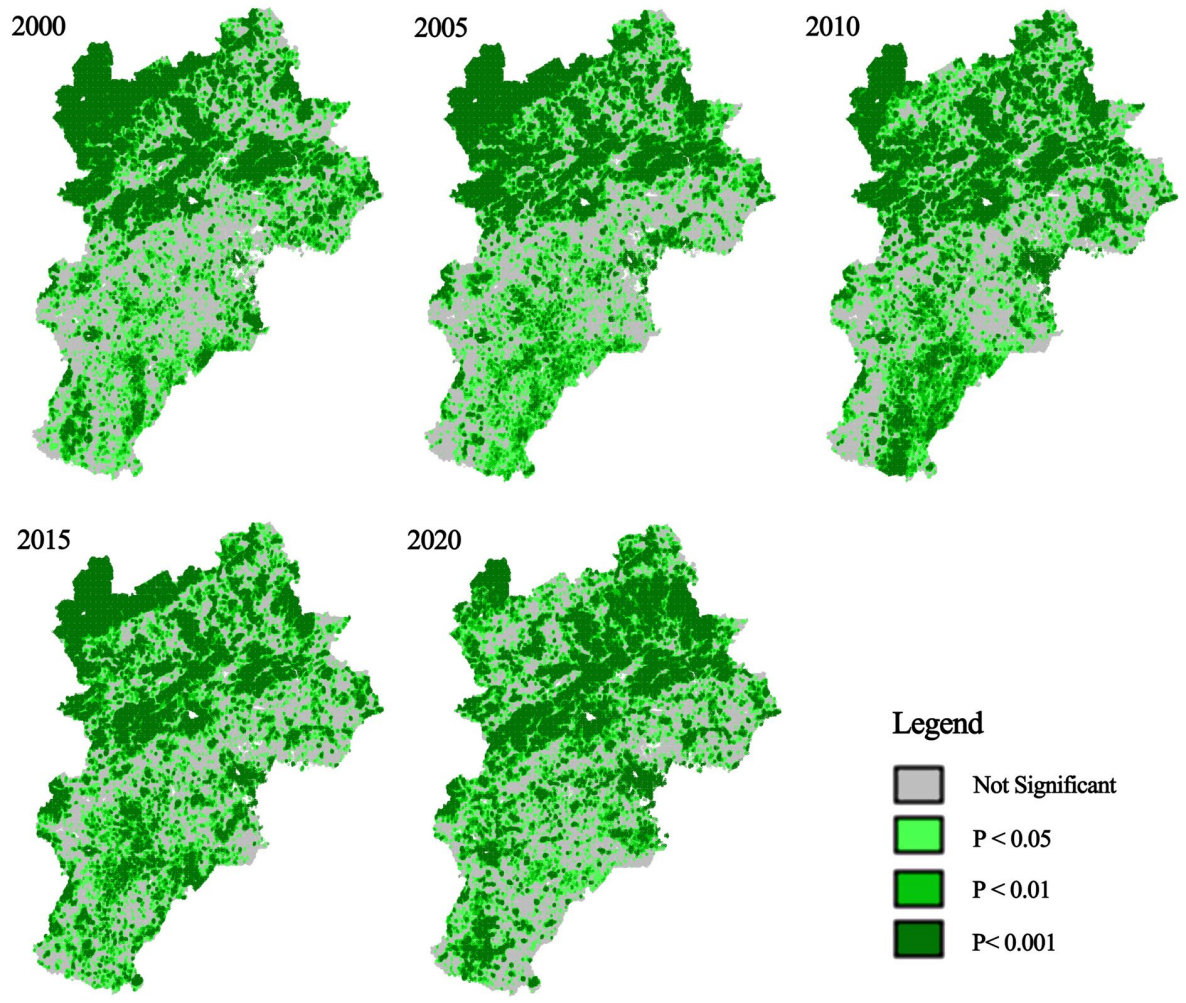
LISA clustering significance plot of RSEI in the BTH urban agglomeration. The figure is generated in GeoDa 1.22.0.4 by applying the clustering significance plot option of the Univariate Local Moran’s I tool to RSEI data sampled in ArcGIS 10.8.

To further explore the spatial distribution traits and clustering phenomena of the RSEI in the BTH urban agglomeration, Moran’s I index was used via using Geoda^51,56^. A total of 279,217 grid cells (1 km × 1 km) were extracted based on the size of the remote sensing image pixels. As shown in Figs. 6, 7 and 8, from 2000 to 2020, the global Moran’s I index values were 0.8679, 0.8754, 0.8647, 0.8743, and 0.8350, respectively, indicating a significant positive spatial autocorrelation. These results were statistically significant at the 99% confidence level. From 2000 to 2005, the H–H regions were mainly concentrated in the northeastern part of the region, with a discontinuous distribution in the southeastern part, whereas the L-L regions with poorer RSEI were primarily concentrated in the northwestern part. The H–H regions showed an expanding trend, whereas the L-L regions exhibited a decreasing trend, with no significant changes in other areas. From 2010 to 2015, the H–H regions remained concentrated in the northeastern and southern regions. The distribution in the southern part expanded from isolated points to continuous patches, whereas the L-L regions were still concentrated in the northwestern and some eastern areas, with no significant changes in other regions. In 2020, the H–H regions were concentrated in the northern region, particularly in the Bashang Plateau, and showed signs of shrinking. The L-L regions were concentrated in the southeastern plains and exhibited an expansion trend, with no significant changes in other areas.

These findings suggest that the spatial distribution of the RSEI in the region is highly clustered, with significant spatial autocorrelation and dynamic changes over the study period.

### PLS-SEM analysis

To ensure the robustness of the PLS-SEM model developed in this study, a Variance Inflation Factor (VIF) test was performed, with the results displayed in Table 3. The VIF was employed to evaluate the degree of multicollinearity among the 12 variables. The test findings indicated that all variables had VIF values below three, suggesting the absence of significant multicollinearity issues^57^.

**Table 3:** VIF for each indicator.

Indicators	VIF	2000	2005	2010	2015	2020
Climate	Precipitation	1.416	1.192	1.326	1.175	1.111
	Evapotranspiration	1.416	1.192	1.326	1.175	1.111
Terrain	Elevation	1.378	1.375	1.374	1.368	1.367
	Slope	1.378	1.375	1.374	1.368	1.367
Urbanization	Nightlight	1.278	1.262	1.253	1.234	1.153
	Population	1.278	1.262	1.253	1.234	1.153
Soil	Sand	1.071	1.071	1.070	1.071	1.070
	SOC	1.071	1.071	1.070	1.071	1.070
Biology	GPP	1.181	1.223	1.227	1.359	1.328
	AGB	1.181	1.223	1.227	1.359	1.328

**Fig. 9:**
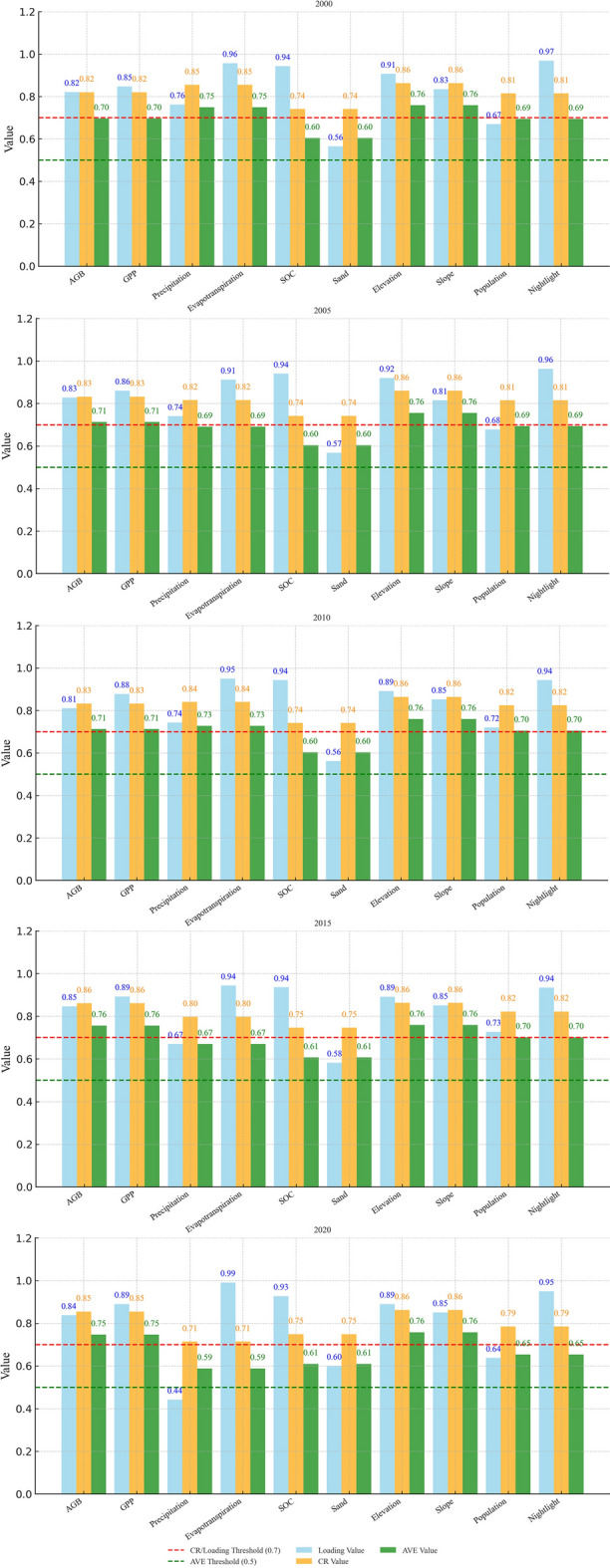
CR and AVE evaluation of PLS-SEM. The figure is created using Python 3.12.6 (https://www.python.org/downloads/release/python-3126/), and the original data comes from the analysis results of the PLS-SEM model.

The path validity test results of the model are presented in Table S1, with all outcomes meeting the significance threshold. The model’s reliability and validity were also assessed^58^. Typically, a Composite Reliability (CR) value of more than 0.7 usually points to acceptable internal consistency, while an Average Variance Extracted (AVE) value above 0.5 demonstrates good convergent validity^59^. As shown in Fig. 9, all variables exhibit CR values above 0.7 and AVE values greater than 0.5, confirming the suitability of the data and the model’s reliability.

**Fig. 10:**
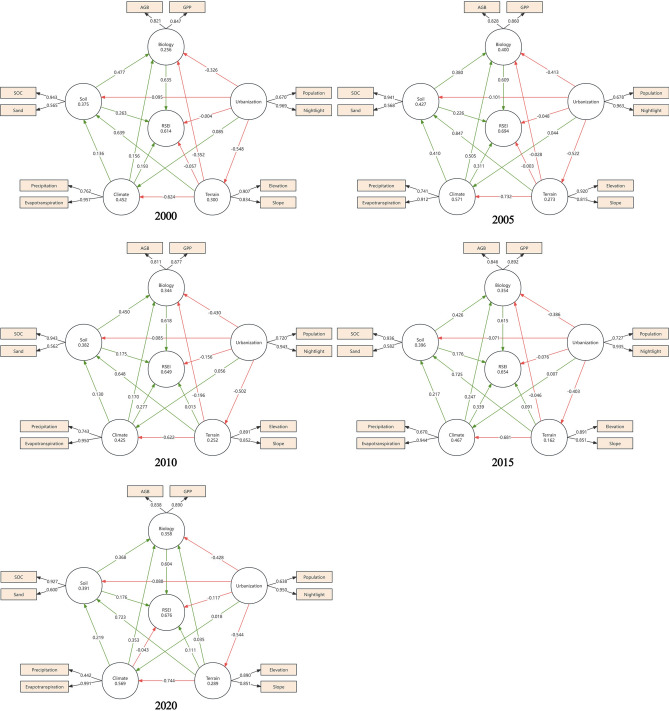
PLS-SEM plot. The figure is generated using SmartPLS 3.2.9 (https://www.smartpls.com/).

As shown in Fig. 10, the PLS-SEM constructed in this study reveals the diverse effects of climate, terrain, urbanization, soil, and biological factors on the RSEI in the BTH urban agglomeration. The model’s explanatory power (R-squared) for the years 2000, 2005, 2010, 2015, and 2020 were 0.614, 0.694, 0.649, 0.654, and 0.676, respectively. These results indicated that the five latent variables effectively explained the formation mechanism of the RSEI. Soil and biological factors positively impacted the RSEI from 2000 to 2020. Among these, biological factors had the most significant effect, with the highest explanatory power observed in 2000 (0.635) and the lowest in 2020 (0.604). Climate factors positively influenced the RSEI from 2000 to 2015 but exhibited a negative impact in 2020. The maximum positive effect occurred in 2015 (0.339), while the smallest negative value was in 2020 (-0.043). Urbanization factors consistently showed negative explanatory power for the RSEI throughout the study period, indicating a negative influence. The greatest negative effect occurred in 2010 (−0.156), and the smallest negative impact was observed in 2000 (-0.004). These findings suggest that while natural factors such as terrain, soil, and biological factors tend to improve ecological quality, climatic factors exhibit a more dynamic influence, and urbanization consistently hinders ecological conditions. The effect of terrain factors increased steadily from −0.057 in 2000 to 0.111 in 2020. During the period from 2000 to 2005, terrain had a slight negative impact on the RSEI. However, starting in 2010, this influence shifted to a positive effect and continued to strengthen thereafter.

## Discussion

Overall, between 2000 and 2020, the RSEI of the BTH urban agglomeration experienced a fluctuating but overall declining trend, with relatively higher values in 2000 and 2010 and lower values in 2005 and 2015. This conclusion is consistent with that of Zhai et al. (2016)^60^. Between 2000 and 2005, the RSEI in the Beijing-Tianjin-Hebei region declined by approximately 10%. During this period, as an industrial base, Zhangjiakou witnessed a marked expansion of areas characterized by poor ecological quality, while the zones of poor and worst ecological quality within the concentrated development areas of other cities also increased. This trend is attributable to the accelerated industrialization of the region, which resulted in a substantial rise in pollutant emissions. Moreover, the implementation of ecological restoration projects such as the “Returning Farmland to Forests” program and the “Beijing-Tianjin Sandstorm Source Control Project” had not yet produced significant ecological benefits, and severe spring sandstorms further exacerbated environmental degradation. From 2005 to 2010, the RSEI increased by approximately 4%. During this interval, the extent of the poorest ecological zones in Zhangjiakou decreased sharply, likely owing to the completion of Phase I and the commencement of Phase II of the “Beijing-Tianjin Sandstorm Source Control Project,” which yielded pronounced restoration outcomes. Additionally, the most degraded areas in major cities such as Beijing and Tianjin contracted significantly, possibly reflecting the heightened attention to ecological governance prompted by the environmental requirements of the 2008 Beijing Olympic Games^61^. In 2014, Beijing’s hosting of the APEC summit, along with strict traffic restrictions and emissions controls, temporarily improved regional ecological conditions but failed to produce lasting ecological benefits^62,63^. Compared to 2010, the RSEI in 2015 declined by approximately 4%, and the loading vector matrix exhibited a pronounced directional shift. Although the regional coordinated development plan for Beijing-Tianjin-Hebei advanced joint environmental governance with significant achievements in air pollution control, water protection, and ecological restoration, the 2015 RSEI downturn is attributable to unprecedented atmospheric pollution induced by the third strongest El Niño on record, which inflicted severe air quality degradation across the region, and led to a decline in RSEI^60^. Meanwhile, despite less rainfall in spring during the 2015 El Niño event, NDVI continued to rise slightly, increasing from 0.7321 in 2010 to 0.7360 in 2015 (Table S3). This may be attributed to accelerated vegetation development driven by earlier warming, as well as the ecological effects of large-scale afforestation and urban greening projects implemented in the region during this period. Relative to 2015, the RSEI in 2020 increased by about 3%. While the region grappled with multiple episodes of severe pollution during the pandemic and post-pandemic industrial rebound led to increased emissions, consistent with the negative climatic influence identified by the PLS model, the lockdown measures and enhanced regional ecological governance during COVID-19 contributed positively to ecological quality improvements^64–66^.

In this study, biological and soil factors consistently had positive impacts on the eco-environmental quality from 2000 to 2020. The influence of terrain factors likewise shifted from negative to positive and continued to strengthen. During this period, the continuous construction of protective forests and wetland reserves significantly increased vegetation cover and species diversity in the northern part of the BTH region, promoting nutrient cycling and energy flow, thereby maintaining ecological balance. Both AGB and GPP play crucial roles in stabilizing the eco-environmental quality through material cycling and carbon storage. Furthermore, the undulating terrain in the study area, which forms windward slopes, contributes to increased precipitation, favorable water-heat conditions, and supported biodiversity. Terrain also limits urban expansion and reduces human disturbance to the environment, and this restriction has gradually become more prominent as cities have developed. These findings corroborate those of Wang et al. (2024), Adhikari and Hartemink (2016), and Xu et al. (2021), who confirmed the positive contributions of biological, soil, and terrain factors to the ecological environment^67,68^. Specifically, the influence of biological factors decreased from 0.635 in 2000 to 0.604 in 2020, marking a decline of approximately 5%. The impact of soil factors steadily decreased from 0.263 in 2000 to 0.176 in 2020, indicating a reduction of about 50%. This suggests that the positive effects of biological and soil factors have been consistently diminishing, likely due to the disturbances caused by urbanization and changes in land use on these factors^69^. Furthermore, the influence of terrain factors incresed from −0.057 in 2000 to 0.111 in 2020. This indicates a continuous enhancement in the positive impact of topographical factors on ecological environmental quality. This could be attributed to factors such as terrain types and slopes significantly influencing the probability of forest and grassland in the Beijing-Tianjin-Hebei region transitioning into other land types, so as to maintain the quality of the ecological environment in the gradually intense expansion of urbanization^70^. Meanwhile, the restrictive effect of terrain on urban expansion is becoming increasingly obvious.

The study also found that climatic factors exerted a positive influence on ecological quality from 2000 to 2015, with their impact increasing by approximately 76% during this period. However, in 2020, this influence reversed and became negative, representing a nearly twofold decrease compared to 2015. Although the composite indicator of precipitation and evapotranspiration is not directly equivalent to LST, these variables are closely linked in the regional water–energy cycle and land–atmosphere energy balance^71^. Between 2000 and 2015, the climate in northern China was relatively mild, and in particular increased precipitation facilitated vegetation recovery and growth, enhanced soil moisture, and improved overall ecosystem health. Moreover, a series of governmental ecological initiatives, such as afforestation programs, effectively capitalized on these favorable climatic conditions, accelerating environmental restoration and creating a positive feedback loop. From 2005 to 2010, however, the BTH urban agglomeration underwent rapid urbanization and widespread land-use change, which exacerbated the urban heat island effect and disrupted hydrological and thermal regimes, leading to a decline in ecological quality^72,73^. At that time, the region also lacked integrated policy frameworks to sustain ecological security improvements, further contributing to environmental degradation^74,75^. From 2010 onward, LST became the dominant factor affecting RSEI, with the Chinese government’s policies, such as “Grain for Green,” the “Beijing-Tianjin Sand Source Control Project (Phase II),” and “Pollution Gas Emission Control,” significantly improving near-surface temperatures and promoting ecological restoration. This finding is consistent with that of Xu et al. (2021), who used the vegetation index-biomass and Net Primary Productivity (NPP) approaches to assess the ecological quality of the BTH urban agglomeration. However, Xu et al. (2021) observed a continuous increase in eco-environmental quality from 2000 to 2010, which differs from the decline observed in the present study from 2000 to 2005. This discrepancy may stem from the findings of Xu et al. (2021), who focused solely on vegetation factors and excluded other variables. Additionally, the PLS-SEM in this study showed that climate negatively impacted eco-environmental quality in 2020^76^. Specifically, the PLS-SEM climate pathway coefficient shifted from positive to negative in 2020, indicating that the contribution of climate to ecological quality changed from facilitative to suppressive. Such changes can be attributed to a series of extreme weather events in the region, including multiple episodes of severe air pollution, serious droughts, and heat waves (China Meteorological Administration, 2021), which collectively imposed significant environmental shocks^77^.

Moreover, this study found that urbanization factors negatively impacted the eco-environmental quality from 2000 to 2020. Urbanization has led to the expansion of urban areas, increased construction land, soil degradation, habitat fragmentation, and species loss. Moreover, continuous population growth has resulted in increased environmental pollution and resource and energy consumption. Therefore, land use changes induced by rapid urbanization have contributed to environmental deterioration, which is consistent with the findings of Du and Huang (2017)^78^. Specifically, the negative impacts of urbanization were relatively mild from 2000 to 2005, at −0.004 and -0.048 respectively, sharply rising to −0.156 in 2010, more than a threefold increase from 2005. However, these figures incresed to −0.111 in 2020, with reduction in magnitude of 41%, indicating a sustained marginal decrease in the adverse effects of urbanization, a shift potentially linked to regional development policies. From 2000 to 2005, China was experiencing a slowdown in growth as it transitioned from a planned economy to a market economy, with the impetus for urbanization in the BTH region not yet fully realized. However, after 2010, the region accelerated urbanization processes by driving economic growth through manufacturing development, thereby accentuating the negative repercussions of urbanization, aligning with the findings of Zhang et al. (2022)^79^. The “National Urban System Planning (2006–2020)” issued in 2010 explicitly identified the BTH urban agglomeration as a key urban cluster. The “Outline of the Development Plan for the Beijing-Tianjin-Hebei Coordinated Development” released in 2015 formally established intensive urbanization, environmental governance, and industrial transfer as development goals, promoting the harmonious urbanization development in the BTH region. This may have led to a sustained marginal decline in the negative effects of urbanization, consistent with the conclusions of Zhao et al. (2024)^80^.

In this study, the overall ranking of the factors affecting the RSEI in the BTH urban agglomeration was biological factors > climatic factors > soil factors > terrain factors > urbanization factors. In contrast, Zhang et al. (2022) ranked the effects on RSEI in the Changsha-Zhuzhou-Xiangtan (CZT) urban agglomeration as follows: terrain factors > urbanization factors > soil factors > climate factors^81^. In this study, biological factors, including AGB and GPP, had the most significant effects on the RSEI, highlighting their central role in eco-environmental quality changes, which is consistent with the findings of Raihan (2023)^82^. The higher total impact of climatic factors in this study compared to that of Zhang et al. (2022) can be attributed to the temperate monsoon climate of the BTH urban agglomeration, which has a more pronounced climate response than the subtropical monsoon climate of the CZT region. The BTH region benefits from significant climatic variation and high biodiversity. Therefore, the temperate climate in the BTH urban agglomeration responds more notably to climatic factors. Conversely, the effects of soil, terrain, and urbanization factors in this study were relatively lower than those reported by Zhang et al. (2022), likely due to regional climate and ecological differences^81^.

Nonetheless, this study has certain limitations. Firstly, although using PC_1_ from PCA method to assign weights in the construction of the RSEI has been shown to be applicable in the BTH region, previous research suggests that the PCA method may lead to information loss or introduce subjective weighting. Therefore, it may not be suitable for all study areas. Future research will explore alternative objective weighting methods to improve the robustness and adaptability of RSEI applications. Secondly, this study employs 1 km resolution remote sensing imagery, which is sufficient to capture macro-scale urban expansion and intensity changes at the provincial level. However, it is insufficient for capturing fine-scale variations in ecological quality within urban neighborhoods. To address this, future studies will incorporate higher-resolution data, such as Sentinel-2 imagery, which offers improved spatial detail and spectral richness, enabling more refined assessments of urban ecological conditions^83^.

## Conclusion and outlook

This study uses GEE to calculate RSEI and applies PLS-SEM to analyze the trends in eco-environmental quality changes in the BTH urban agglomeration. It also investigates the mechanisms through which biological, climatic, soil, topographical, and urbanization factors influence these changes. From 2000 to 2020, the RSEI in the BTH urban agglomeration displayed a downward fluctuating trend. Specifically, the area with the highest RSEI decreased from 7.99% in 2000 to 1.20% in 2020, while areas with poor and extremely poor RSEI levels fluctuated upwards, increasing from 4.80% in 2000 to 15.89% in 2020. NDVI emerged as the dominant driver of RSEI in the BTH urban agglomeration, with its contribution to the principal components peaking in 2000 and exhibiting a fluctuating downward trend thereafter until 2020. In 2015, the principal component structure underwent a notable shift, as LST transitioned to a positive variable in PC_1_ and the cosine similarity declined significantly. These reversals indicate a fundamental change in the region’s underlying ecological regulation mechanisms. Over the period from 2000 to 2020, the factors influencing eco-environmental quality in the BTH urban agglomeration were ranked as follows: biological factors > climatic factors > soil factors > topographical factors > urbanization factors, with biological factors being crucial in preserving eco-environmental quality. The spatial layout of RSEI in the BTH urban agglomeration also exhibited significant clustering patterns, with H–H areas mainly located in the northern Bashang Plateau and L-L areas concentrated in the southeastern plains. Over time, L-L areas expanded from isolated spots to more continuous regions.

This study offers suggestions based on the problems emerging in the BTH urban agglomeration: (1) Strengthen coordinated ecological restoration across the BTH region by expanding protective forests and ecological corridors through large-scale afforestation and wetland rehabilitation, thereby boosting regional vegetation cover and biodiversity. (2) Enhance joint air-pollution prevention and control by deepening collaboration between provincial meteorological services and environmental protection agencies to develop a unified early-warning system for cross-boundary pollution events. (3) Improve sustainable land-resource management by undertaking targeted remediation of key watersheds and contaminated sites, and by implementing soil-conservation measures that increase soil organic matter and water-retention capacity. (4) Scale up ecological compensation in mountainous areas through terraced-field improvements to reduce soil erosion and optimize local microclimates. (5) Strictly enforce the red lines for arable land, ecological protection, and urban-development boundaries by optimizing urban spatial layouts and enhancing the efficiency of urban land use to balance growth with conservation.

## Supplementary Information

Supplementary Information.

## Data Availability

The data sets used in the current study are available from the corresponding author on reasonable request.
