# Supplementary material for: Spatiotemporal evolution and driving factors of eco-environmental quality in the Beijing-Tianjin-Hebei urban agglomeration in China
Source: Sci Rep. 2025 Jul 15;15:25631. doi: 10.1038/s41598-025-11751-y (PMC12263879; doi:10.1038/s41598-025-11751-y)
Supplement: Supplementary file 1 — Supplementary Information. [file 41598_2025_11751_MOESM1_ESM.docx]

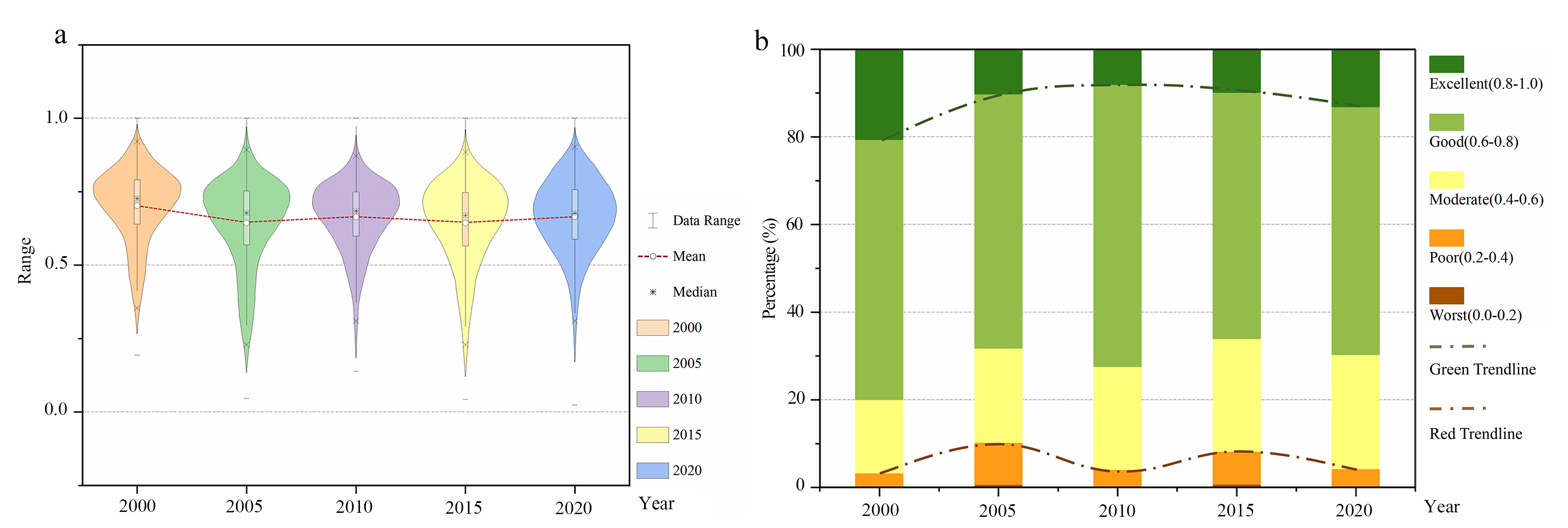


**Supplementary Figure 3.** Box plot of RSEI for BTH urban agglomeration and percentage of regions at each level (2000-2020).

**Supplementary Legend:** a: Box plot of RSEI for BTH urban agglomeration, which is used to observe the distribution of RSEI and the change of average value. b: Percentage of regions at each level of eco-environmental quality, the green trend line shows the proportion of areas with excellent ecological quality, and the red trend line shows the proportion of areas with worst and poor ecological quality.

**Supplementary Figure 4.** Spatial distribution of RSEI in BTH urban agglomeration (2000-2020).

**Supplementary Legend:** These maps are calculated by Google Earth Engine, the original code is attached in the chapter of the data source.

**Supplementary Figure 5.** RSEI transfer in the BTH urban agglomeration (2000-2020).

**Supplementary Legend:** According to the spatial resolution of the MODIS sensor, a sampling grid of 1km×1km was divided to obtain 277,200 effective sampling grid cells, and the results of RSEI evaluation were spatial-sampled. The generated transfer graph can intuitively present the spatial variation characteristics of RSEI level of each sampling point.

**Supplementary Figure 8.** Scatter plot of RSEI Moran’s Index for the BTH urban agglomeration.

**Supplementary Legend:** Scatter plot, LISA clustering and its significance plot were obtained by Geoda (https://geodacenter.github.io/download.html).


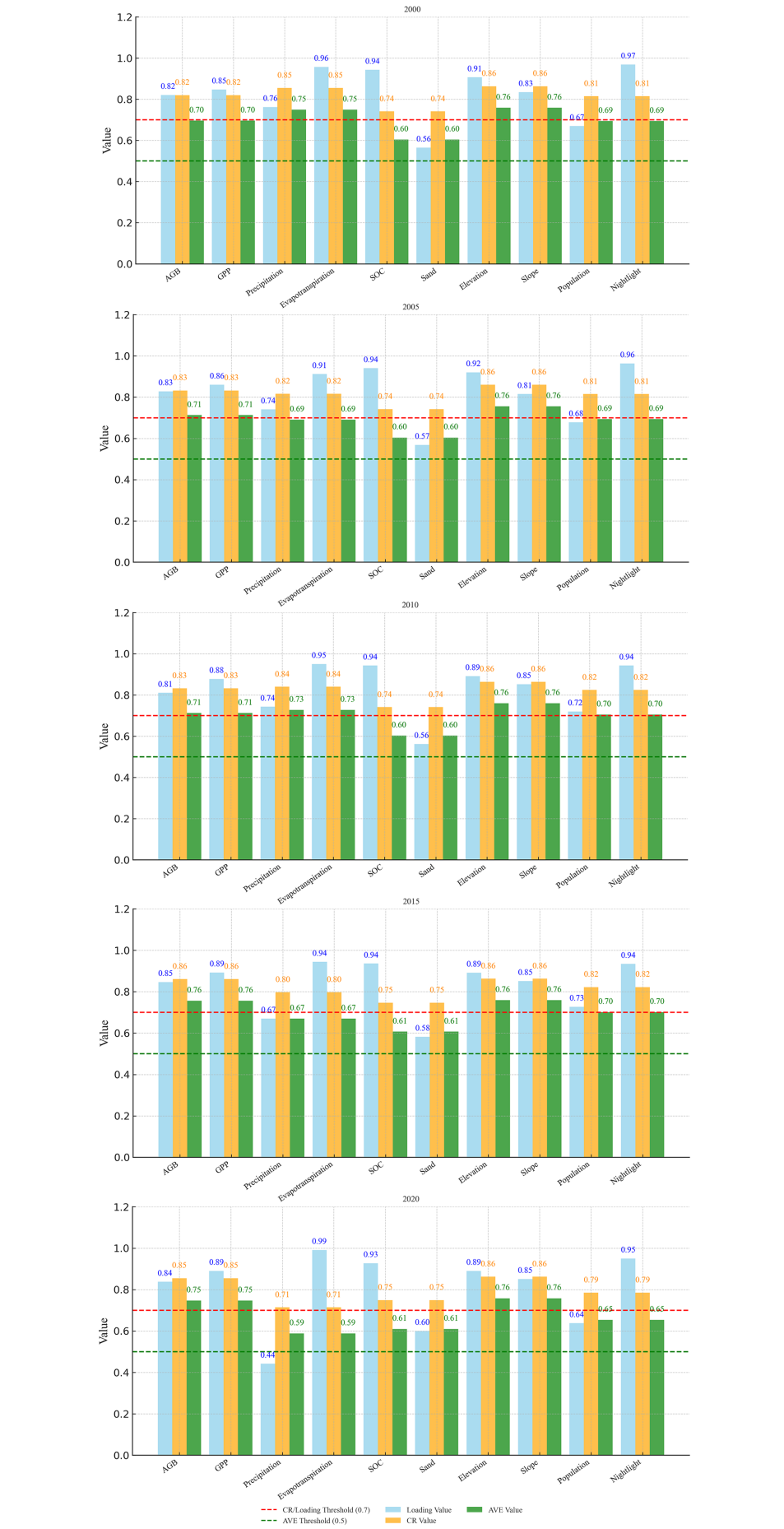


**Supplementary Figure 9.** CR and AVE evaluation of PLS-SEM.

**Supplementary Legend:** All indicators demonstrated acceptable reliability (CR > 0.7) and validity (AVE > 0.5). And the Loading Value represented the contribution to RSEI.

| **Year** | **Path** | **Indicators** | **Path Coefficient** |
| --- | --- | --- | --- |
| 2000 | Biology | RSEI | 0.635 |
|  | Climate | Biology | 0.156 |
|  |  | Soil | 0.136 |
|  |  | RSEI | 0.193 |
|  | Soil | Biology | 0.477 |
|  |  | RSEI | 0.263 |
|  | Terrain | Biology | -0.352 |
|  |  | Climate | -0.624 |
|  |  | Soil | 0.639 |
|  |  | RSEI | -0.057 |
|  | Urbanization | Biology | -0.326 |
|  |  | Climate | 0.082 |
|  |  | Soil | -0.095 |
|  |  | Terrain | -0.548 |
|  |  | RSEI | -0.004 |
| 2005 | Biology | RSEI | 0.609 |
|  | Climate | Biology | 0.505 |
|  |  | Soil | 0.410 |
|  |  | RSEI | 0.311 |
|  | Soil | Biology | 0.380 |
|  |  | RSEI | 0.226 |
|  | Terrain | Biology | -0.028 |
|  |  | Climate | -0.732 |
|  |  | Soil | 0.847 |
|  |  | RSEI | -0.003 |
|  | Urbanization | Biology | -0.413 |
|  |  | Climate | 0.044 |
|  |  | Soil | -0.048 |
|  |  | Terrain | -0.101 |
|  |  | RSEI | -0.526 |
| 2010 | Biology | RSEI | 0.618 |
|  | Climate | Biology | 0.170 |
|  |  | Soil | 0.130 |
|  |  | RSEI | 0.277 |
|  | Soil | Biology | 0.450 |
|  |  | RSEI | 0.175 |
|  | Terrain | Biology | -0.196 |
|  |  | Climate | -0.622 |
|  |  | Soil | 0.648 |
|  |  | RSEI | 0.013 |
|  | Urbanization | Biology | -0.430 |
|  |  | Climate | 0.056 |
|  |  | Soil | -0.085 |
|  |  | Terrain | -0.502 |
|  |  | RSEI | -0.156 |
| 2015 | Biology | RSEI | 0.615 |
|  | Climate | Biology | 0.247 |
|  |  | Soil | 0.217 |
|  |  | RSEI | 0.339 |
|  | Soil | Biology | 0.426 |
|  |  | RSEI | 0.176 |
|  | Terrain | Biology | -0.046 |
|  |  | Climate | -0.681 |
|  |  | Soil | 0.725 |
|  |  | RSEI | 0.091 |
|  | Urbanization | Biology | -0.386 |
|  |  | Climate | 0.007 |
|  |  | Soil | -0.071 |
|  |  | Terrain | -0.403 |
|  |  | RSEI | -0.076 |
| 2020 | Biology | RSEI | 0.604 |
|  | Climate | Biology | 0.353 |
|  |  | Soil | 0.219 |
|  |  | RSEI | -0.043 |
|  | Soil | Biology | 0.368 |
|  |  | RSEI | 0.176 |
|  | Terrain | Biology | 0.035 |
|  |  | Climate | -0.744 |
|  |  | Soil | 0.723 |
|  |  | RSEI | 0.111 |
|  | Urbanization | Biology | -0.428 |
|  |  | Climate | 0.018 |
|  |  | Soil | -0.088 |
|  |  | Terrain | -0.538 |
|  |  | RSEI | -0.117 |

**Supplementary Table S1.** Path validity of PLS-SEM

| **Indicators** | | **2000** | **2005** | **2010** | **2015** | **2020** |
| --- | --- | --- | --- | --- | --- | --- |
| Cosine Similarity | PC1 | 1 | 0.8500 | 0.9591 | 0.7810 | 0.9461 |
|  | PC2 | 1 | -0.8814 | 0.7912 | -0.4593 | 0.5956 |
|  | PC3 | 1 | 0.9642 | 0.8147 | -0.5343 | -0.6363 |
|  | PC4 | 1 | -0.995 | -0.9898 | -0.9952 | -0.9964 |
| EV(PC1) | | 0.0135 | 0.0175 | 0.0140 | 0.0171 | 0.0139 |

**Supplementary Table S2.** Cosine Similarity of the Independent PCA result.

| **year** | **LST_mean** | **NDBSI_mean** | **NDVI_mean** | **WET_mean** |
| --- | --- | --- | --- | --- |
| 2000 | 0.648253 | 0.858993 | 0.708984 | 0.460797 |
| 2005 | 0.628050 | 0.860507 | 0.729155 | 0.485826 |
| 2010 | 0.595179 | 0.855892 | 0.732126 | 0.513834 |
| 2015 | 0.603398 | 0.857858 | 0.736017 | 0.493005 |
| 2020 | 0.611616 | 0.857022 | 0.778610 | 0.517315 |

**Supplementary Table S3.** The average values of each RSEI index in summer.
